# Exploring Submerged Forward Osmosis for Water Recovery and Pre-Concentration of Wastewater before Anaerobic Digestion: A Pilot Scale Study

**DOI:** 10.3390/membranes9080097

**Published:** 2019-08-05

**Authors:** Federico Ferrari, Maite Pijuan, Ignasi Rodriguez-Roda, Gaetan Blandin

**Affiliations:** 1 Catalan Institute for Water Research (ICRA), Emili Grahit Street, 101, 17003 Girona, Spain; 2 Campus Montilivi, University of Girona, 17003 Girona, Spain; 3 LEQUiA, Laboratory of Chemical and Environmental Engineering, Campus Montilivi, University of Girona, 17071 Girona, Spain; 4 Eurecat, Centre Tecnològic de Catalunya. Plaça de la Ciència, 2. 08242 Manresa, Spain

**Keywords:** forward osmosis, real municipal wastewater, pilot scale, submerged membrane, anaerobic digestion, water reuse, wastewater concentration

## Abstract

Applying forward osmosis directly on raw municipal wastewater is of high interest for the simultaneous production of a high quality permeate for water reuse and pre-concentrating wastewater for anaerobic digestion. This pilot scale study investigates, for the first time, the feasibility of concentrating real raw municipal wastewater using a submerged plate and frame forward osmosis module (0.34 m^2^) to reach 70% water recovery. Membrane performance, fouling behavior, and effective concentration of wastewater compounds were examined. Two different draw solutions (NaCl and MgCl_2_), operating either with constant draw concentration or in batch with draw dilution over time, were evaluated. Impact of gas sparging on fouling and external concentration polarization was also assessed. Water fluxes up to 15 L m^−2^ h^−1^ were obtained with clean water and 35 g NaCl/L as feed and draw solution, respectively. When using real wastewater, submerged forward osmosis proved to be resilient to clogging, demonstrating its suitability for application on municipal or other complex wastewater; operating with 11.7 g NaCl/L constant draw solution, water and reverse salt fluxes up to 5.1 ± 1.0 L m^−2^ h^−1^ and 4.8 ± 2.6 g m^−2^ h^−1^ were observed, respectively. Positively, total and soluble chemical oxygen demand concentration factors of 2.47 ± 0.15 and 1.86 ± 0.08, respectively, were achieved, making wastewater more suitable for anaerobic treatment.

## 1. Introduction

The increasing demand of water and a crisis in water quality due to the combined effects of the increasing urbanization and climate change are forcing many countries to consider significant policy changes and expensive measures for water reclamation and wastewater (WW) treatment [1]. Two of the solutions being considered to tackle this challenge are lowering potable water consumption and reusing WW from various sources, such as municipal WW. For this reason, during the last decade, the classical concept of WW treatment and pollution removal has evolved towards treatment lines and processes that promote the recovery of water, energy, and chemical compounds [2]. Among others, forward osmosis (FO) and anaerobic digestion (AD) are promising WW treatment technologies to produce, on one side, high quality water appropriate for reuse—for example, in irrigation—and on the other side, to recover energy in the form of methane-rich biogas, respectively [3,4,5]. FO relies on a semi-permeable membrane and an osmotic gradient driving force, and has the advantage of moderate fouling propensity and easier fouling removal when compared to pressure driven membranes [6]. On the other hand, AD is a technology used for the treatment of organic wastes already implemented worldwide—especially for the treatment of primary and secondary sludge produced in WW treatment plants (WWTPs) to degrade organic matter and produce biogas.

An integration of these two technologies has attracted increasing interest in recent years, with several studies focusing on various combinations. For example, FO was used to treat the centrate of an anaerobic digester [7] and the permeate produced from an anaerobic membrane bioreactor (AnMBR) [8,9] for nutrient recovery. Coupling AD and FO was also investigated through anaerobic osmotic membrane bioreactors by replacing the microfiltration or nanofiltration membrane module of an AnMBR with an FO membrane [10,11]. Although promising, this configuration presents several challenges due to its high fouling propensity and lower reversibility, which were shown to result in a significant decrease of the water flux (Jw). Also, to tackle the salt accumulation due to reverse solute flux (RSF) and the consequent inhibition of the anaerobic biomass, the addition of microfiltration and the use of expensive salts as draw solutes were required previously [12,13,14]. As such, incorporating an FO membrane within the biological reactor appears challenging.

An alternative configuration is the use of FO to pre-concentrate WW before subsequent AD, as described in Ansari et al. in 2017 [15]. In this review, the authors mentioned higher biogas production, recovery of nutrients, and increased lifespan of the membranes, thanks to lower membrane fouling propensity, higher fouling reversibility, and lower membrane degradation. The main benefit of this configuration was the smaller digester volume needed to anaerobically treat the concentrated WW, leading to lower energy needs for the digester heating.

All previously mentioned, FO studies have remained at lab scale and have used side stream membrane modules, which are far from full-scale application. An FO operation (as in other membrane systems) can be implemented in submerged mode, when the membrane is placed in the feed tank or in side stream mode, and the membrane module is located outside the tank, typically in a cross flow configuration. The performance of a submerged FO (Sub-FO) configuration was compared with that of a side-stream configuration in a study conducted by Morrow in 2018 [16] for an osmotic membrane bioreactor (OMBR). The two configurations resulted in similar steady-state water fluxes for both draw solute concentrations tested (35 and 100 g NaCl/L), but the RSF was higher for the submerged module, while the greater energy consumption for the side-stream configuration could be due to the additional recirculation pumping needed. High capital and operation costs due to the high crossflow velocities (CFVs) made the crossflow membrane system too expensive, especially for municipal WW treatment [17]. In our recent work on a small pilot scale Sub-FO, we demonstrated significant progress in terms of module design and operation in the scope of an OMBR leading to low and recoverable fouling, a high rejection rate, and a possibility to treat complex streams—as well as to reach higher fluxes [18,19], which can be of high interest in the context of an FO-AD hybrid system.

This present study examines a novel, up-scaled, and newly designed Sub-FO pilot scale system for raw municipal WW concentration. In the first step, the system was optimized using: (1) Tap water, (2) tap water with salt (to mimic the concentration of salt in a typical municipal WW), and (3) real municipal WW. Then, tests were conducted to concentrate municipal raw WW to reach 70% water recovery. The effect of different gas sparging procedures on membrane performance and concentrated WW characteristics was also assessed.

## 2. Materials and Methods 

### 2.1. Pilot Set-up and Operating Conditions

**Figure 1:**
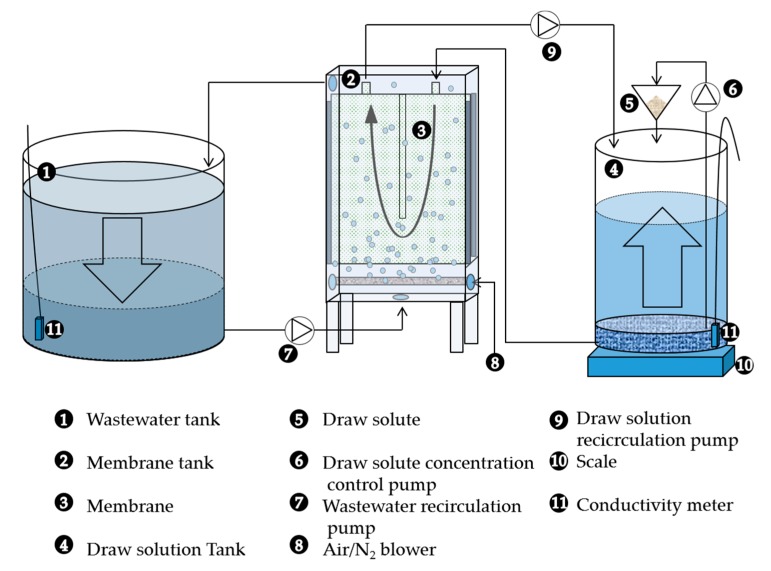
Schematic diagram of the forward osmosis (FO) bench-scale set-up. Arrows in the tank describe changes in volumes during the FO concentration process.

The pilot set-up was composed of three units: (1) A storage tank for the WW, (2) an FO module, and (3) a tank for the draw solution (Figure 1). The FO module was a U-shaped plate and frame module, assembled as described in [19] using a Kubota plate (Kubota, Osaka, Japan) and a Toray thin film composite (TFC) membrane sheet (Toray, Tokyo, Japan) (A, B and S data are 8.9 ± 0.14 L m^−2^ h^−1^ bar^−1^, 5.68 ± 0.14 L m^−2^ h^−1^, and 466 × 10^−6^ m [20]), with a surface area of 0.336 m^2^. The FO module was vertically positioned in the reactor, consisting of a 9 L methacrylate structure equipped with a gas sparging system. The space between the membrane and the structure was 0.5 cm to control particle deposition; fouling and external concentration polarization (ECP) were mitigated through gas scouring (gas bubbles on membrane surface). WW entered from the bottom of the FO module with a flow rate of 1.8 L/min (WW 0.72 m/min cross flow velocity).

Jw (L/m^2^ h) was calculated by using the following formula:

(1) $$J_{w}\;=\frac{\left(\mathrm{Change}\;\mathrm{is}\;\mathrm{DS}\;\mathrm{volume}\;\left(L\right)\right)}{\left(A_{m}\left(m^{2}\right)\text{ × }∆t\;\left(h\right)\right)}$$

where A_m_ is the effective membrane surface area (m^2^), and Δt is the operation time (h).

RSF was determined using the following formula:

(2) $$\mathrm{RSF}\;=\left(\frac{\left(\mathrm{Change}\;\mathrm{is}\;\mathrm{DS}\;\mathrm{conductivity}\;\left(\mathrm{mS}/\mathrm{cm}\right)\text{ × }\mathrm{FC}\;(g/\left(\mathrm{mS}/\mathrm{cm}\right)\right)}{\left(A_{m}\left(m^{2}\right)\text{ × }∆t\;\left(h\right)\right)}\right)$$

where FC is the calibration factor for the draw solute concentration versus conductivity (FC_MgCl__2_ = 0.46 g/(mS/cm), FC_NaCl_= 0.51 g/(mS/cm)) obtained using calibration curves.

Water recovery was defined as:

(3) $$\mathrm{Water}\;\mathrm{Recovery}\;=\;\frac{\mathrm{Permeated}\;\mathrm{volume}\;(L)}{\mathrm{Initial}\;\mathrm{feed}\;\mathrm{volume}\;(L)}\text{ × }100$$

### 2.2. Filtration Tests

Four different set of tests were conducted. The first three sets of tests were conducted to evaluate the impact of operating conditions, i.e., draw solution type, CFV, air sparging, feed conductivity, and draw concentration on water flux. In the fourth set of tests, the optimum operating configuration was chosen and tested with real WW. A description of each set of tests is presented below.

Impact of draw solution type on water permeation flux: The first set of tests was conducted using 60 L of tap water as feed solution and 2 L of 35 g/L of salt as draw solution. NaCl and MgCl_2_ were tested to study the dependence of water flux on the salt used as draw solute. 

Impact of CFV and air sparging on water flux: The second set of tests was done using 60 L of tap water as a feed solution and 2 L of a solution containing 70 g/L of sea salt (>99.4% NaCl), provided by Vicens I Batllori S.L. (Banyoles, Spain), as a draw solution, letting it dilute as the test progressed. Tests lasted for 2 h. Two CFVs (0.27 and 0.72 m/min) were tested by changing the section of the methacrylate structure, both with and without air sparging. 

Impact of feed water salinity and draw concentration: The third set of tests was conducted using 113 L of tap water with a conductivity of 0.6 mS/cm as a feed solution. Tests were stopped when they reached 62% water recovery (the draw solution recirculation pump stopped once the draw solution reached the volume associated with 62% water recovery, measured through a scale connected to a programmable logic controller (PLC)). Two draw solution configurations were tested: (1) 30 L with an initial salt concentration of 35 g/L (which was diluted during the filtration time), and (2) 20 L with an initial salt concentration of 11.7 g/L (which was kept constant by adding salt during the experiment by recirculating the draw solution through a filter containing salt (Figure 1) with a recirculation pump, controlled via a conductivity probe (Crison instruments, Barcelona, Spain) as detailed in Sauchelli et al.[21]). Both configurations resulted in the same amount of salt utilization at the end of the concentration test. Conductivities of the feed and draw solutions were continuously monitored. Two different feed solutions were used: Tap water and saline water with a conductivity of 1.9 mS/cm, as to mimic ions and conductivity from areal municipal WW. The saline water composition used was (in mg/L): 183 of NH_4_Cl, 280 of NaHCO_3_, 750 of MgSO_4_·7H_2_O, 118 of CaCl_2_·2H_2_O. 

Impact of real municipal WW: In the fourth set of tests, 50 L of municipal WW collected from the inlet of a local WWTP (Quart WWTP, Spain) and prefiltered with a 5 mm mesh sieve was used as a feed solution, and 10 L of sea salt solution (11.7 g/L) was used as a draw solution. Tests finished once 70% water recovery was reached. Three different gas sparging procedures were studied: (1) continuous air sparging, (2) intermittent nitrogen gas sparging (1min on/15min off), and (3) absence of gas sparging. The operating temperature was set at 17 °C. After each test, 2 L of distilled water was recirculated inside the membrane and purged to manually clean the membrane surface, as described in Blandin et al. [22,23]. WW composition at the beginning and at the end of the tests was analyzed to determine any possible chemical oxygen demand (COD) degradation in real WW. Triplicates were conducted for each test.

### 2.3. Assessment of Membrane Integrity and Performance

A two hours flux test was carried out to assess membrane integrity before each test. For the third set of tests, the same flux test was conducted after each test, and also after the cleaning procedure, to respectively quantify the fouling effect after each WW concentration process and the cleaning efficiency. For this purpose, 25 L of tap water and 20 L of sea salt solution (35 g/L) were used as feed and draw solutions, respectively. Conductivity of the feed solution was monitored to quantify the RSF, and draw solution conductivity was not controlled. Osmotic backwash (OBW) cleaning was also conducted after the assessment of the membrane performance. To this end, 20 L of salt solution (35 g/L) was placed in the WW tank and 25 L of tap water in the draw solution tank; the FO unit was operated for two hours in OBW mode. The whole system was then rinsed using tap water.

### 2.4. Chemical Analysis

Municipal WW (feed) and draw solution samples were taken and immediately filtered through 0.2 µm Millipore filters before and after each test conducted with municipal WW for subsequent analysis. Phosphate (PO_4_^3−^), sulfate (SO_4_^2−^), ammonium (NH_4_^+^), potassium (K^+^), magnesium (Mg^2+^), and calcium (Ca^2+^) were analyzed via ion chromatography (ICS5000, DIONEX) (Thermo scientific, Waltham, Massachusetts, US). Total COD and soluble COD concentrations were also measured using test kits (Hach Lange, Dusseldorf, Germany). Total organic carbon (TOC) concentration was analyzed by a TOC analyzer (TOC-V_CSH_, Shimadzu) (Shimatdzu, Kyoto, Japan).

## 3. Results and Discussion 

### 3.1. Tests with Tap Water

**Figure 2:**
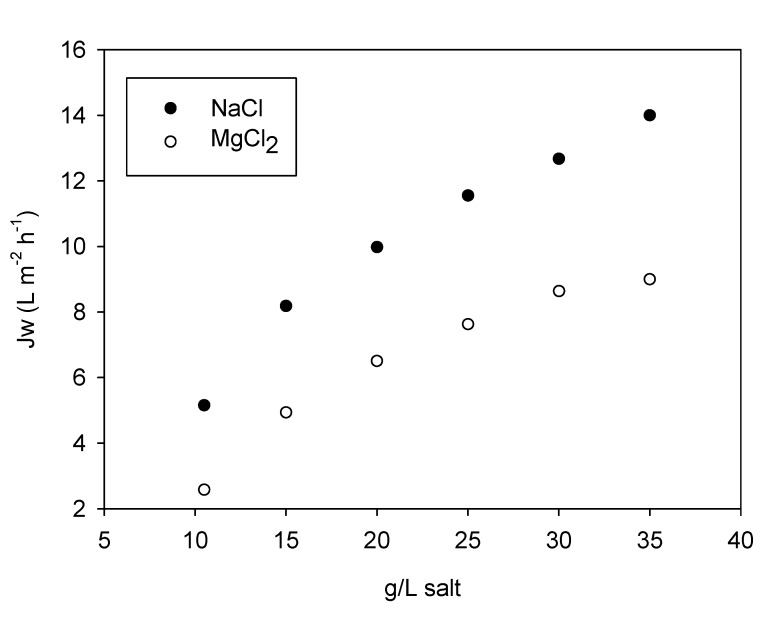
Water flux dependence on the draw solute concentration and the type of salt used.

FO tests with tap water and an initial draw solution concentration of 35 g/L of salt (dilution overtime) were conducted to study the impact of the type of salt used as draw solute (MgCl_2_ and sea salt) on water flux. Figure 2 shows the results obtained in these tests.

Water fluxes were generally high (starting from 14.1 and 8.2 L m^−2^ h^−1^ for 35 g/L of sea salt and MgCl_2_, respectively) and in line with results obtained in a previous study [19] with smaller FO modules (0.05 m^2^ of surface area), proving the good functioning of the Sub-FO plate design even at larger scale. The lower Jw using MgCl_2_ led to longer filtration times as compared to the test with sea salt (42.9 and 25.6 h, respectively). Surprisingly, the use of MgCl_2_ as draw solute was also accompanied by a higher RSF, averaging 7.6 g m^−2^ h^−1^, compared to sea salt with 6.7 g m^−2^ h^−1^, leading to final salt concentrations in the feed solution of 3.16 and 2.24 g/L for MgCl_2_ and NaCl, respectively. The resulted Js/Jw ratio (0.95) was the same obtained in a previous study [18] with a smaller TFC Submerged FO module after 8 days of permeation in an OMBR and using NaCl as draw solute. The original salt present in the feed accounted for 26% and 40% of the final concentration for MgCl_2_ and NaCl, respectively. With this membrane module, NaCl resulted in a more appropriate draw solute compared to MgCl_2_, not only thanks to the higher water flux obtained and its lower price, but also the lower final salinity in the concentrated feed solution, which must be considered for its potential inhibitory effect in a downstream biological system.

**Figure 3:**
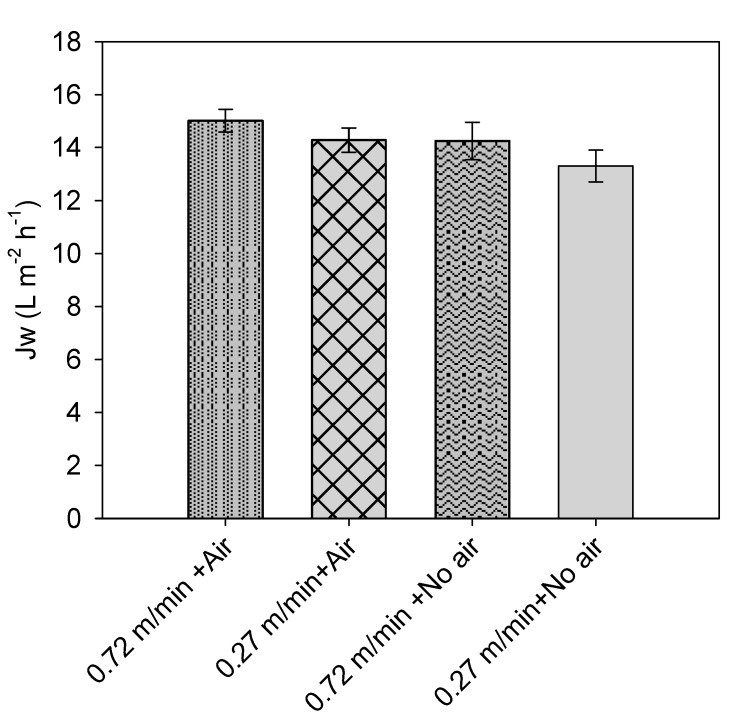
Impact of crossflow velocity (CFV) and an air sparging application on the average initial water flux (first 10 min of operation).

Figure 3 shows results of the second set of tests conducted to assess the effect of CFV (0.27 and 0.72 m/min) and air sparging (with/without) on Jw.

**Figure 4:**
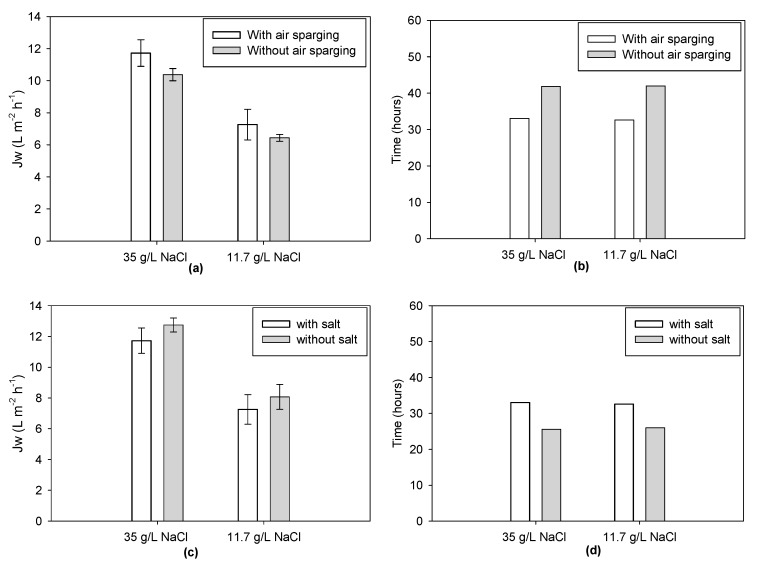
Average water fluxes during the first 2 h of each test (**a**,**c**); total filtration time for 35 g/L of draw solute (without re-concentration) and for the 11.7 g/L constant draw solute concentration under different conditions (**b**,**d**).

All tested configurations resulted in slightly different Jw after 10 min, but remained mostly within the interval of uncertainty; only the last configuration (no air sparging, low CFV) showed a significantly lower flux. On the other hand, operating the system at 0.72 m/min CFV with air sparging resulted in the highest permeation flux. The two configurations 0.72 m/min CFV without air sparging, and 0.27 m/min CFV with air sparging—had similar results with lower average fluxes. Overall, these results indicate that the higher the shear stress (higher CFV and air sparging), the lower the ECP. However, the effects remained quite mild in the conditions tested and in the first minutes of the filtration tests. Further tests were performed during a longer period (i.e., 2 h); the impact of feed salinity and the application of air sparging for two types of draw solute operations (dilution over time or constant concentration) is shown in Figure 4.

When looking at 35 g/L draw solution tests (dilution over time), the application of air sparging resulted in 11.42 ± 0.04 L m^−2^ h^−1^ initial water flux, which was higher compared to the same conditions but without air sparging (11.4% higher for 11.7 g NaCl/L and 11.5% for 35 g NaCl/L). Similar observations could be made with a constant draw operation, confirming the benefits of air sparging in mitigating ECP. In both cases, longer filtration time was needed to achieve the desired concentration rate when no air sparging was implemented. Operating at a constant draw solute concentration of 11.7 g/L of sea salt throughout the test led to 38.01 ± 0.20% lower initial Jw compared to the higher initial draw solute concentration (35 g/L of sea salt, without re-concentration). However, the final test duration to reach the desired water recovery was similar for both configurations. This is due to the sharper flux decrease when the system is operated at a higher initial draw concentration but without re-concentration. With time, permeation flux decreases to very low levels and filtration time is extended. Since both configurations use the same amount of salt and similar filtration time, a system operated with draw re-concentration would be preferred, allowing operation at lower and constant flux and providing lower fouling rate and higher process stability.

Finally, both initial flux and filtration time varied depending on the initial salinity of the feed solution. When feed solution mimicking the WW salinity was used, test duration was 22.8% higher than when using tap water. This is due to the lower initial osmotic pressure driving force as a result of the higher salinity of the feed, which is reinforced by the enhanced salinity increase in the feed overtime and the higher overall feed salinity at the end of the concentration process.

### 3.2. Tests with Municipal WW

**Figure 5:**
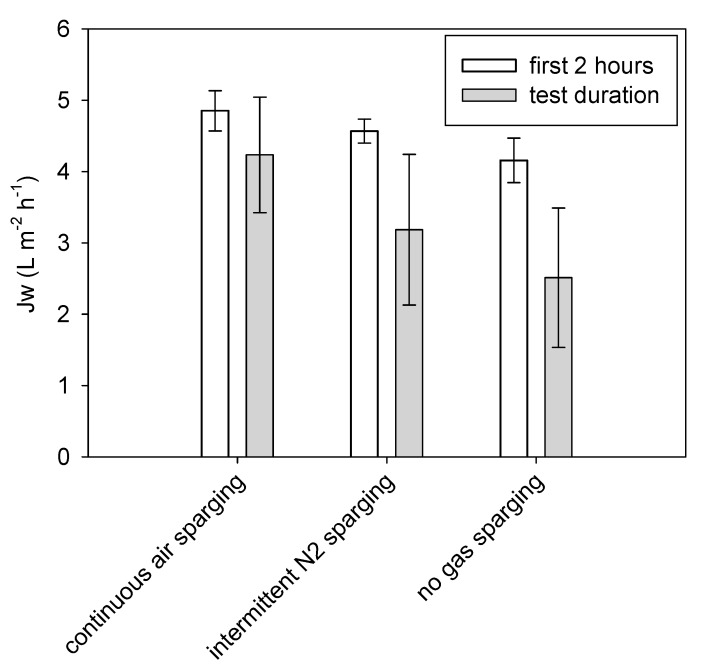
Average water flux with standard deviation during the first 2 h of each test and during the whole test duration, under different gas sparging conditions.

Three tests with real municipal WW were conducted to study the effect of three different gas sparging procedures (continuous air sparging, intermittent N_2_ sparging 1 min on/15 min off, and absence of gas sparging) on Jw. Test duration, COD concentration, and degradation, as well as ion concentrations, were monitored. Figure 5 shows the average Jw of the first 2 h of each test (initial flux) and average Jw to reach 70% water recovery.

Continuous air sparging and intermittent N_2_ sparging led to lower flux declines, and implied 40.1% and 17.4% less time to reach 70% water recovery when compared to the configuration without gas sparging (lasting 23.9, 33.0 and 39.9 h, respectively). The benefits of the application of gas sparging were clear from the beginning of the tests (Figure 5) and increased throughout their duration, due to the effect of gas sparging on ECP, as well as on fouling control. RSF was similar for the three sets of tests, being 4.8 ± 2.6, 4.2 ± 2.2, and 3.9 ± 2.4 g m^−2^ h^−1^, and leading to a final salt concentration in the WW of 5.1 ± 1.7, 6.4 ± 1.2, and 7.2 ± 0.8 mg/L for continuous air sparging, intermittent N_2_ sparging, and no gas sparging, respectively. The highest final salt concentration obtained under the lowest gas sparging intensity can be attributed to the longer test duration necessary to reach 70% water recovery, leading to more salt passage.

**Figure 6:**
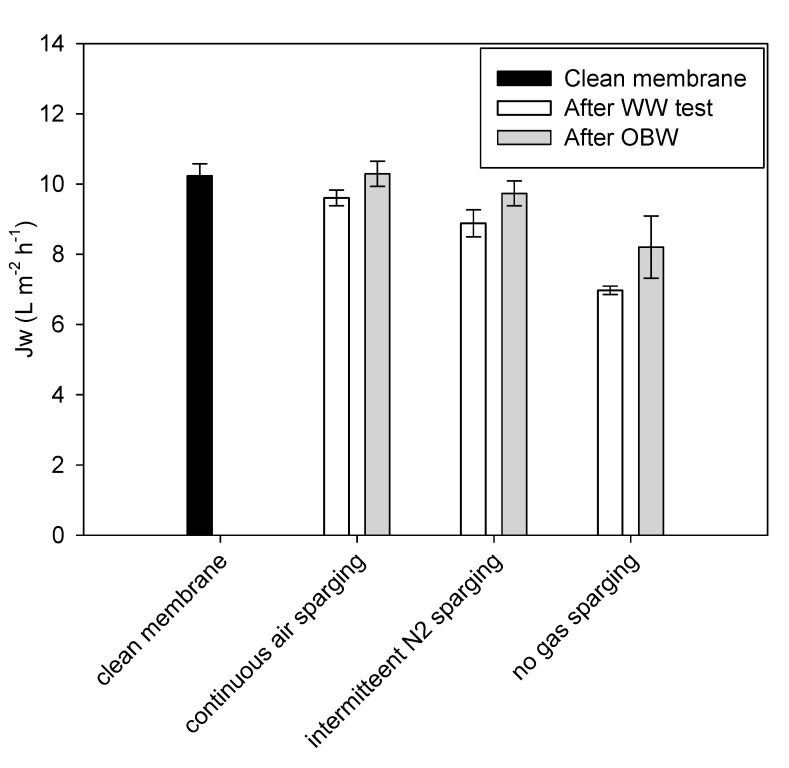
Comparison of water fluxes results of the flux test with clean membrane, after WW tests and after osmotic backwash (OBW). Effect of gas sparging procedure on Jw. (Tests conducted with 35 g/L salt in the draw solution)

The effect of the gas sparging procedure on membrane fouling was also assessed by comparing the flux before (clean membrane) and after each WW test, using tap water as feed solution and with continuous air sparging (Figure 6).

After the filtration batches, a Jw decline of 5.7%, 12.4%, and 31.3% was observed with continuous air sparging, intermittent N_2_sparging, and no gas sparging, respectively, confirming that gas sparging (even if intermittent) has a strong effect on fouling mitigation. On the other hand, the strong flux decline when no gas sparging was applied confirms that operation without gas sparging is challenging. Additionally, OBW allowed for flux recovery in all cases. Full recovery of the initial flux was achieved when OBW was applied after continuous gas sparging and nearly full recovery (95.1%) when applied after intermittent gas sparging. These results indicate that a sustainable procedure for operation and cleaning was found. However, it has to be tested for extended filtration times and repeated batches. For the no gas sparging batch, OBD did not allow for full recovery of the initial flux (only 80%), confirming that operating with gas sparging is preferable. These results are encouraging, especially when air sparging is implemented, since it confirms the low fouling potential of Sub-FO and easy cleaning even when treating very complex streams such as raw WW. This is further reinforced by the fact that no problems related to clogging were encountered during the duration of the tests, and that no membrane degradation was detected. These results show that pre-treatment of the WW, which for other membrane configurations such as a crossflow module is considered as an important procedure to decrease the likelihood of clogging and degradation issues, can be very limited in the case of a submerged plate and frame module. This would further simplify and make economically more attractive this technology, and, in the case of an anaerobic treatment of the concentrated WW, it would mean that no COD would be lost in the pre-treatment stages, maximizing its concentration and subsequent conversion into biogas. On the other hand, due to the interaction between gas bubble and the surfactants present in the WW, foaming was observed when operating the system with gas sparging compared to tests in the absence of gas sparging.

#### 3.2.1. COD Concentration during the Filtration Batch

**Figure 7:**
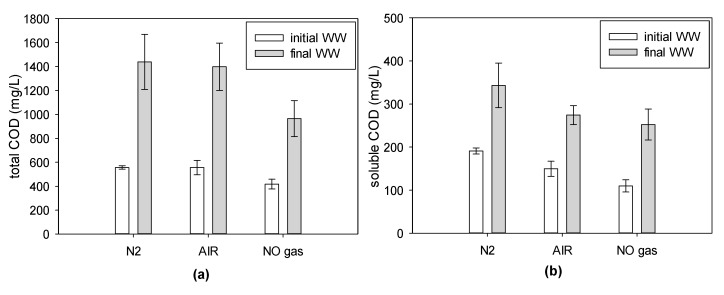
Initial and final WW COD concentration: Total COD (**a**); soluble COD (**b**).

A 70% water recovery (extraction) from WW via FO implies the production of a concentrated stream rich in COD, which can be treated in an anaerobic reactor to recover biogas. However, effective concentration of COD needed to be assessed, therefore total and soluble COD and TOC were measured in the WW at the beginning of the test and in the concentrate stream at the end of each test (Figure 7).

In all tests, independently from the gas sparging configuration, a concentration factor (CF) of 2.47 ± 0.15 and 1.86 ± 0.08 was achieved for total and soluble COD, respectively. These values represent 75 ± 4% and 56 ± 2% of the theoretical CF (3.33), implying a loss of COD was present in the WW. Several hypotheses were formulated to explain the loss, namely: (1) Passage in the draw solution, (2) degradation during the filtration due the presence of air, and (3) natural degradation in the WW due to filtration time. The TOC present in the final draw solution (COD analysis of the draw solution was not reliable due to the interference of the high salt concentration) accounted for 3.7 ± 0.4% of the initial TOC (80.1 ± 7.0 mg TOC/L) and, therefore, potential loss due to lack of membrane rejection remains very limited. The impact of air (vs. N_2_) sparging did not show a significant difference and COD losses were observed in both cases. To investigate if COD loss could be attributed to the WW degradation at 17 °C temperature during the filtration time, 100 mL of WW was left outside the WW tank and COD was monitored at the beginning and at the end of each test. Results showed that 12% and 36% of the total and soluble COD present in the WW, respectively, was lost in the storage tank. Considering these losses and calculating the theoretical final concentration with the COD concentrations of the biodegraded samples, the real final WW concentration represented 85.5 ± 5.8% and 88.3 ± 6.6% of the theoretical values for the total and soluble COD, respectively. Thus, it indicates that the main cause of COD loss during filtration is the natural raw WW degradation during the time of filtration. Other (minimal) COD losses might have been due to deposition in the reactor (estimated to be up to 3% by collecting and analyzing leftover WW sludge in the bottom of the reactor) and to foaming. Overall, the final COD concentration of the concentrated WW was close to 1500 mg/L, which is considered the lowest COD concentration limit that is needed to produce enough quantities of methane to heat the WW without an external fuel source, and therefore confirms the use of FO for effective concentration of organic matter. Further improvement of the process can be achieved to improve COD recovery by minimizing dead zones and foaming, and reducing the filtration time.

#### 3.2.2. Ion Concentrations during Filtration Batch

**Figure 8:**
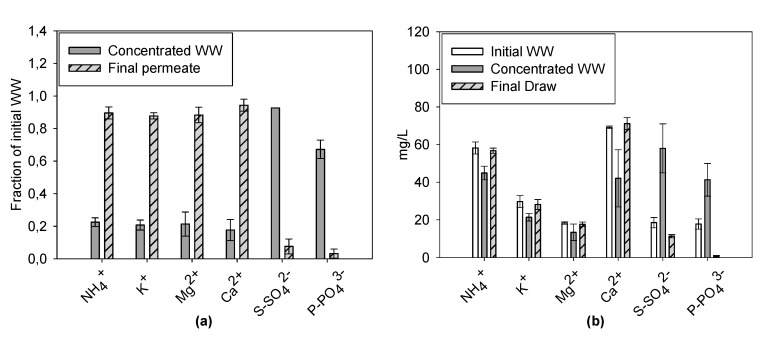
Average ion mass balance with standard deviation at the end of the test with real WW (**a**). Average ion concentrations with standard deviation of the test with real WW in the original WW, concentrated WW, and final permeate at the end of the test (**b**).

Analysis of the ions present in the draw and feed solution before and after the test were performed, showing high differences in the rejection of cations and anions (Figure 8).

The concentration of cations (NH_4_^+^, K^+^, Mg^2+^, and Ca^2+^) in the WW after each test was lower compared to the original concentration, implying that the retention of cations by the process was negative, with the majority (always more than 80%) ending in the draw solution (Figure 8a). The anions (SO_4_^2−^ and PO_4_^3−^) followed the opposite trend, with a low fraction passing to the draw solution (always lower than 10%), resulting in a higher concentration, up to 3.3 fold for SO_4_^2−^ (same as theoretical CF) and up to 2.6 for PO_4_^3−^, compared to values of the original WW. These results are similar to the results obtained by Xue et al. [24]. In this article, different membrane materials and orientations were tested to study ion rejection during an FO process. Interestingly, while for Cellulose Triacetate (CTA) membranes high removals were obtained for every ion species studied, in the case of TFC membranes, different results were obtained depending on the orientation (with the active layer facing the feed solution or the draw solution). In the configuration used in our study (active layer facing the feed solution), negative retention was obtained for ammonia rejection, while 90% retention was obtained for phosphate. With the active layer facing the draw solution, the phosphate removal remained the same as for the other configuration, while ammonia rejection increased to 50%. This phenomenon can be explained with two hypotheses, namely: (1) Greater ammonium permeability of the TFC membrane compared to the CTA one (one order of magnitude higher); and (2) high negative zeta potential of the TFC membranes, which is at a similar level to that of a cation exchange membrane (cation exchange like mechanism). Other articles with TFC membranes provide the same interpretation [21,25], but one study [14] explains the poor cation retention due to the Donnan equilibrium and the use of sea salt as a draw solute. The high concentration of NaCl in the draw solution and the higher diffusion coefficient of Na^+^ compared to Cl^−^ leads to a higher amount of Na^+^ in the feed solution, creating a charge imbalance between the feed and draw solutions. To compensate for this imbalance, more cations diffuse from the feed solution to the draw solution. For further understanding on TFC membrane selectivity, and to confirm whether Donnan equilibrium—in combination with the use of NaCl as a draw solute—is the mechanism which leads to poor cation rejection, future studies are needed to show that electroneutrality is maintained in the final WW solution and the draw solution. The concentrated WW turned out to be more suitable for an anaerobic treatment than before concentration as a result of its higher COD concentration (>1500 mg/L). As a side effect, a high concentration of SO_4_^2−^ (Figure 8b) was also achieved, resulting in the COD: SO_4_^2−^ ratio being slightly lower compared to the original WW (27.5 and 23.3 for raw and concentrated WW, respectively). The NH_4_^+^ concentration in the concentrated WW was similar to the original WW and remained lower than 200 mg/L, a concentration at which ammonia is considered to be inhibitory for AD [26]. For the development of FO in combination with AD, future studies should focus on draw solute, such as biodegradable organic solutes, and improvements of FO membrane materials to reduce inhibition of the anaerobic biomass, due to the RSF, and to increase the Jw [27].

## 4. Conclusions

The present study showed the performance of a pilot scale submerged plate and frame FO module for the treatment of real municipal WW, and its concentration for a subsequent anaerobic treatment. The water fluxes obtained were high and in-line with previous results obtained with a lab scale module. Applying continuous air sparging helped maintain a high water flux and a lower fouling propensity and WW salinity. Compared to the initial WW, the final concentrated WW proved to be more suitable for an anaerobic treatment because of the increase of COD concentration (always higher than 1200 mg/L). This work has also demonstrated that for this sub-FO module, there were neither clogging nor degradation issues, making it more economically reasonable and more attractive for a scale up without the need of WW pre-treatment. However, further studies are needed to assess fouling propensity and membrane integrity over prolonged use. Also, a study comparing the performance of a continuous AnMBR, treating raw WW and concentrated WW, should be conducted to assess the difference in reactor performance, not only in terms of energy demand, but also in terms of the quality of the produced biogas and permeate.
